# Genetic Diversity of Torch Ginger (*Etlingera elatior*) Germplasm Revealed by ISSR and SSR Markers

**DOI:** 10.1155/2019/5904804

**Published:** 2019-05-06

**Authors:** Nor Asiah Ismail, M. Y. Rafii, T. M. M. Mahmud, M. M. Hanafi, Gous Miah

**Affiliations:** ^1^Department of Crop Science, Faculty of Agriculture, Universiti Putra Malaysia, 43400 UPM Serdang, Selangor, Malaysia; ^2^Malaysia Agricultural Research and Development Institute, 43400 Serdang, Selangor, Malaysia; ^3^Institute of Tropical Agriculture and Food Security, Universiti Putra Malaysia, 43400 UPM Serdang, Selangor, Malaysia; ^4^Department of Land Management, Faculty of Agriculture, Universiti Putra Malaysia, 43400 UPM Serdang, Selangor, Malaysia

## Abstract

Fifty-seven accessions of torch ginger (*Etlingera elatior*) collected from seven states in Peninsular Malaysia were evaluated for their molecular characteristics using ISSR and SSR markers to assess the pattern of genetic diversity and association among the characteristics. Diversity study through molecular characterization showed that high variability existed among the 57 torch ginger accessions. ISSR and SSR molecular markers revealed the presence of high genetic variability among the torch ginger accessions. The combination of different molecular markers offered reliable and convincing information about the genetic diversity of torch ginger germplasm. This study found that SSR marker was more informative compared to ISSR marker in determination of gene diversity, polymorphic information content (PIC), and heterozygosity in this population. SSR also revealed high ability in evaluating diversity levels, genetic structure, and relationships of torch ginger due to their codominance and rich allelic diversity. High level of genetic diversity discovered by SSR markers showed the effectiveness of this marker to detect the polymorphism in this germplasm collection.

## 1. Introduction

Genetic resources are prerequisite for varietal development. The usefulness of genetic resources depends on the levels of their diversity. Torch ginger (*Etlingera elatior*), a member of Zingiberaceae, is a multifunctional crop but no information is available on its genetic resources. Torch ginger is commonly propagated by rhizomes (asexually). It takes about 12 months after planting to start flowering but a longer time is needed when seeds are used [1]. It is a perennial herb with closely grouped pseudostems reaching height of 3-4 m. The inflorescence is torch-like, borne on erect stalks up to 1.5 m tall arising from fleshy underground rhizomes [2, 3].

Vegetative crop like torch ginger had alternative methods for improvement such as the combination of mutation breeding with tissue culture and molecular marker. Yunus et al. [4] managed to establish a protocol for regeneration of shoot and mutation induction for torch ginger. The combination of several techniques could accelerate the breeding plan for torch ginger.

Since torch ginger is recognized as a medicinal plant, much of the available documented information focused on its biochemical aspect such as antioxidant [5, 6], antibacterial [7, 8], and antifungal activities [9, 10]. There is a need to conduct studies to evaluate the genetic diversity of torch ginger for breeding and conservation purposes. The limited research on characterization at DNA level has hindered the improvement of cultivated torch ginger through molecular breeding.

Evaluation of genetic variation and patterns of population/species genetic diversity allows us to draw specific methodology for its breeding, fast adaptation, and conservation. Various marker-based techniques are accessible for the identification and characterization of genetic variation. Among these, molecular marker is more accepted because it overcomes many of the constraints of morphological and biochemical markers, because it is not influenced by the environment and growing phases of the plant. Every molecular marker has its own qualities; therefore, it must be chosen properly based on its informativeness and ease of genotyping [11].

The evaluation of genetic diversity could be done within and between populations at molecular level by using different techniques like allozymes or DNA analysis [12]. Throughout the years, application of molecular markers has become popular in the diversity assessment of species at the DNA level. PCR-based molecular markers were widely applied for classification, genetic linkage mapping, phylogenetic study, and population analysis [13]. The identification of plant genotypes by using molecular techniques is more effective compared to morphological markers because it permits direct access to the hereditary material [14]. On the other hand, indistinguishable plants can be genotypically different and, therefore, variants of a given cultivar cannot be easily detected by phenotypic assessment. In rice, simple sequence repeat (SSR) markers have been commonly used in genetic diversity studies, which helps to establish the relationship among the individuals even with little number of markers [15]. In other plant species such as pitaya [16], faba bean [17], soybean [18], and sunflower [19], SSR markers were applied for genetic diversity study.

The use of molecular markers for studying genetic diversity was reported for certain members of Zingiberaceae [20–23] but there are very few reports on torch ginger. Until now, 56 genomic SSR markers and 17 EST-SSR have been developed for* Curcuma longa *[24–26]. Meanwhile, only eight genomic SSRs have been reported in* Zingiber officinale *[27]. The only presented report on torch ginger SSR-based diversity analysis is the study by Goncalves [28] to select potential genotypes for cut flower. Fortes* et al.* [29] screened the effective primers for molecular characterization of torch ginger using random amplified polymorphic DNA (RAPD) markers. Since torch ginger is a very poorly studied crop and its molecular information is limited, it is imperative to know the genetic diversity among the different torch ginger accessions in Malaysia.

Therefore, molecular markers were used to determine the genetic diversity and the relatedness of* E. elatior* accessions on Malaysian Agricultural Research and Development Institute (MARDI) germplasm collection. The present study assessed the molecular diversity in 57 accessions of torch ginger using intersimple sequence repeat (ISSR) and simple sequence repeat (SSR) primers. Genetic diversity studies in* E. elatior* will be helpful to resolve the identity of accessions (collected from different localities) and to analyze the intraspecific diversity existing among them. Furthermore, the studies will facilitate the process of planning conservation strategies and optimal utilization of the species. On the other hand, the information on torch ginger genetic relationship will support the genebank management and crop improvement programme in the future.

## 2. Materials and Methods

### 2.1. Plant Materials

Fifty-seven accessions of torch ginger (*Etlingera elatior*) germplasm were sampled from MARDI Jerangau, Terengganu, Malaysia (Table 1). Young leaves were harvested and stored at -80°C until DNA extraction.

### 2.2. DNA Extraction and Quantification

DNA was extracted from young leaves following the modified method of Zheng* et al.* [30]. Young leaves tissues were ground to fine powder in the presence of liquid nitrogen using mortar and pestle. The powder was then transferred into 1.5 mL tube until it reaches 0.5 mL mark. The ground sample was mixed well with 800 *μ*l of CTAB extraction buffer [100 mM Tris-HCl (pH 8.0), 20 mM EDTA (pH 8.0), 1.4 M NaCl, and 2% CTAB] and 2 *μ*l *β*-mercapto-ethanol using vortex. An equal volume of chloroform/isoamyl alcohol, CIA (24:1 v/v), was added and the mixture was centrifuged at 14,000 rpm for 30 sec to separate leaf residues. The supernatant was collected into a new tube and the step with CIA was repeated twice. The supernatant was transferred again into new 1.5 mL tube and added with 800 *μ*l absolute ethanol. The mixture was mixed well by gentle shaking and centrifuged at 14,000 rpm for 3 min. After discarding the supernatant, the DNA pellet was washed by adding 1 mL 70% ethanol at -20°C, dried, and dissolved in TE buffer (10 mM Tris, 1 mM EDTA pH 8.0). RNase enzyme (50 *μ*g/mL) was added and incubated at 37°C for 1 hour. For purification, ethanol precipitation was done by adding potassium acetate (5.0 M) with two volumes of isopropanol at -20°C, mixed well 1-2 min by shaking, and centrifuged at 12,000 rpm (4°C) for 10 min. The DNA was precipitated and washed twice by adding 1 mL 70% ethanol at -20°C. The DNA was pelleted by centrifuging at 10,000 rpm for 10 min, dried, and dissolved in 100 *μ*l of TE buffer. The DNA solution was measured to check the concentration and quality with Nanodrop BioPhotometer (Eppendorf, Hamburg, Germany). The quality of DNA extracted was also checked by running the electrophoresis (Bio-Rad, USA) of DNA samples. DNA samples with ratio absorbance 260/280 of more than 1.8 were considered good to proceed for the next steps. DNA template was diluted to final concentration of 60 ng/*μ*l. The DNAs were stored at -20°C until further use.

### 2.3. Polymerase Chain Reaction (PCR) Protocols

#### 2.3.1. Intersimple Sequence Repeats (ISSR)

Out of 100 ISSR primers, 11 sets of primers (Table 2) were chosen based on their ability to detect distinct polymorphic band across all the 57 torch ginger accessions. PCR reaction mixture contained MyTaq Red Mix (MgCl, dNTPs, buffer, and Taq) (Bioline), primer, template DNA, and nuclease-free water. PCR was performed in thermocycler (Eppendorf, Germany). The initial denaturation was set for 3 min at 95°C, followed by 35 cycles of denaturation at 95°C for 30 sec, annealing at 51-55°C for 30 sec, and extension at 72°C for 1 min. Annealing temperature was adjusted depending on the primer used. The amplified products were separated on 1.5% agarose gel with 1×TBE buffer by electrophoresis at 60 V for 150 min. The gels were visualized and acquired under UV light using GelDoc2000 documentation system. Only distinct and reproducible bands produced by ISSR primers were scored as present (1) or absent (0) with all the accessions studied. Binary qualitative data matrix was constructed for further analysis.

#### 2.3.2. Simple Sequence Repeats (SSR)

A total of 21 microsatellite primers developed by Goncalves [28] were initially screened with* E. elatior* DNA for reproducible amplification of 57 accessions. Only six primers (Eela2, Eela4, Eela5, Eela17, Eela18, and Eela21) were able to show amplification on 57 accessions for SSR analysis (Table 3). The remaining primers showed no amplification at all. The amplification reactions were performed in a final volume of 25 *μ*l containing MyTaq Red Mix (MgCl, dNTPs, buffer, and Taq), forward and reverse primers, DNA template, and nuclease-free water. The total amplification cycle was performed in a thermocycler programmed to start at 94°C for 5 min, 10 cycles at 94°C for 1 min, 58°C for 1 min (with a decrease of 1°C per cycle), and 72°C for 1 min plus 30 cycles at 94°C for 40 s, 48°C for 40 s, and 72°C for 1 min, as well as final extension at 72°C for 10 min. The amplification products were separated on Fragment Analyzer and evaluated using the software package ProSize 2.0 (Advanced Analytical, USA). The amplified bands were scored according to the size of DNA ladder (1kb Plus). Each band fragment size was recorded in Microsoft Excel for analysis.

### 2.4. Data Analysis

#### 2.4.1. ISSR Analysis

Primarily, the potential of the marker for estimating genetic variability of* E. elatior* was examined by assessing the marker informativeness through the bands scoring. Primer banding characteristics such as number of total bands (TB), number of polymorphic bands (PB), and percentage of polymorphic bands (PPB) were obtained. In order to analyze the suitability of marker for genetic profile evaluation, the performance of ISSR markers used was measured using three parameters: polymorphic information content (PIC), marker index (MI), and resolving power (RP). The PIC value for each primer was calculated as described by Roldan-Ruiz* et al.* [31]; PIC_*i*_ = 2f_*i*_ (1-f_*i*_), where PIC_*i*_ is the polymorphic information content of the locus* i*, f_i_ is the frequency of the amplified allele, and 1-f_i_ is the frequency of nonamplified allele. The frequency was calculated as the proportion between the number of amplified alleles at each locus and the total number of accessions. The PIC of each primer was calculated using the average PIC value from all loci of each primer. Marker index for each primer was calculated as product of polymorphic information content and effective multiplex ratio according to Varshney* et al.* [32]; MI = PIC x EMR, where EMR is the effective multiplex ratio. Effective multiplex ratio was estimated as EMR =* n* x *β*, where n is the average number of alleles amplified by accession to a specific system marker (multiplex ratio) and *β* is estimated after considering the number of polymorphic loci (PB) and nonpolymorphic loci (MB); *β* = PB/ (PB+MB). Resolving power is the ability of a primer to distinguish genotypes, which was calculated as RP = *∑*Ib, where Ib represents the informative bands [33]. The Ib can be represented into a 0/1 scale by using the following formula: Ib = 1 – (2 x ∣0.5 – p_*i*_∣), where p_*i*_ is the ratio of accessions comprising the* i*th band.

The genotype and allelic frequency data were used to compute the genetic diversity indices: (1) Shannon's information index (I) by Shannon and Weaver [34];* I* = -*∑p*_*i*_ ln* p*_*i*_, where* p*_*i*_ is the allelic frequency of the* i*th allele in question for the specific accession; (2) Nei's genetic diversity (*h*) according to Nei [35];* h* = 1 - *∑p*_*i*_^2^, where pi is the frequency of the ith allele at the locus. Shannon's information index and Nei's gene diversity with other genetic diversity parameters such as the observed number of alleles per locus (na) and the effective number of alleles per locus (ne) were calculated with the aid of the POPGENE software version 1.32 [36].

GenAIEx 6.5 software [37] was utilized to generate the grouped population gene frequencies as well as Nei's [38] genetic distances matrix between the populations. Other parameters that were analyzed include observed number of alleles per locus (N_a_), number of effective alleles per locus (N_e_) = 1/(p^2^ +q^2^), Shannon's information index (SI) = -1(p Ln(p) + q Ln(q)), the expected heterozygosity (H_e_) = 2pq, the unbiased expected heterozygosity (UH_e_) = (2N/(2N-1)) H_e_, and percentage of polymorphic loci (%P).

The binary data matrix was converted into genetic similarity coefficient between pairs of accessions using Dice coefficient by NTSYS-pc version 2.10 [39]. The distances coefficient was used to construct dendrogram using unweighted pair grouped method for arithmetic average (UPGMA). Principle component analysis (PCA) was also done to highlight the resolving power of the ordination.

#### 2.4.2. SSR Analysis

Genetic diversity was evaluated using POWERMARKER Ver. 3.25 [40]. The genetic parameters included Nei's gene diversity and polymorphism information content (PIC). Nei's gene diversity was defined as the probability that two randomly chosen alleles from the population are different. PIC values provide an estimate of the probability of finding polymorphism between two random samples of the germplasm. A genetic similarity between the accessions was measured by the similarity coefficient using NTSYS-pc version 2.10. Cluster analysis was performed to construct dendrogram based on the similarity matrix data using the unweight pair group method (UPGMA) and the SAHN clustering program. The data was also subjected to PCA (principal component analysis) to explore the structure of germplasm collection. The PCA of the 57 torch ginger accessions were assessed by EIGEN module of NTSYS-pc 2.10.

## 3. Results

### 3.1. ISSR Analysis

Eleven primers that displayed clear and reproducible bands were obtained through the screening of a total 100 primers. A total of 197 bands were revealed, with 100% polymorphism and an average of 17.9 loci by primer. The sizes of the amplicons ranged from 290 to 4000 bp. The information on the genetic profile of each accession obtained using the eleven ISSR primers was used to estimate the marker performance through evaluation of four parameters: polymorphic information content (PIC), effective multiplex ratio EMR, marker index (MI), and resolving power (RP) (Table 4). High PIC value was detected for primer UBC888 at 0.50 and low PIC value of 0.32 for three primers, namely, UBC830, UBC855, and UBC891. The average of PIC value per primer, 0.37, was obtained from the 57* E. elatior* accessions. The ISSR effective multiplex ratio (EMR) may be influenced by the fraction of polymorphic loci. The highest EMR (28.73) was detected with the primer UBC888 and the lowest was shown by the primer UBC830 (11.88), with a mean EMR of 15.19 per primer. General usefulness of the system markers used was determined by the calculation of marker index (MI) for each ISSR primer. The highest MI is shown by the primer UBC888 (14.35) and the lowest in the primer UBC830 (3.83) with a mean MI of 5.86 per primer. The resolving power (RP) is the ability of a primer to differentiate between genotypes. The average RP was 8.72 per ISSR primer. The highest RP value was detected with the primer UBC885 (12.72) and the lowest with the primer UBC811 (5.51). Primer UBC817 showed the highest number of effective alleles per locus (1.54) and the lowest alleles were observed in UBC811 with the value of 1.36 (Table 5). Nei's index ranged from 0.22 to 0.31 with the maximum value in UBC817 and the lowest in UBC811 with the mean value of 0.26, while Shannon's information index ranged between 0.35 and 0.47. Primer UBC817 showed the highest value (0.47) and the lowest value (0.35) was detected in UBC811 with the average value of 0.41.

#### 3.1.1. Genetic Diversity in* E. elatior* Populations

The 57 accessions were divided into seven major populations based on the geographical locations from which they were collected. The percentage of polymorphic loci for each population ranged from 0.24% (Melaka) to 0.79% (Terengganu) with average of 0.52% (Table 6). Genetic variability among the population as revealed by expected heterozygosity (H_e_) showed that Pahang population possessed greater level of variability with value of 0.20 as compared to other populations which ranged from 0.10 to 0.20. Shannon's information index among populations ranged from 0.14 to 0.31 with the highest value observed in the Pahang population. The pairwise population matrix of Nei's genetic distance of seven states was estimated (Table 7). The maximum genetic distance (0.08) was detected between Melaka-Perak, Kelantan-Kedah, and Kelantan-Pahang population pairs. The least genetic distance (0.02) was found in Terengganu-Perak pair.

#### 3.1.2. Cluster Analysis of ISSR

**Figure 1:**
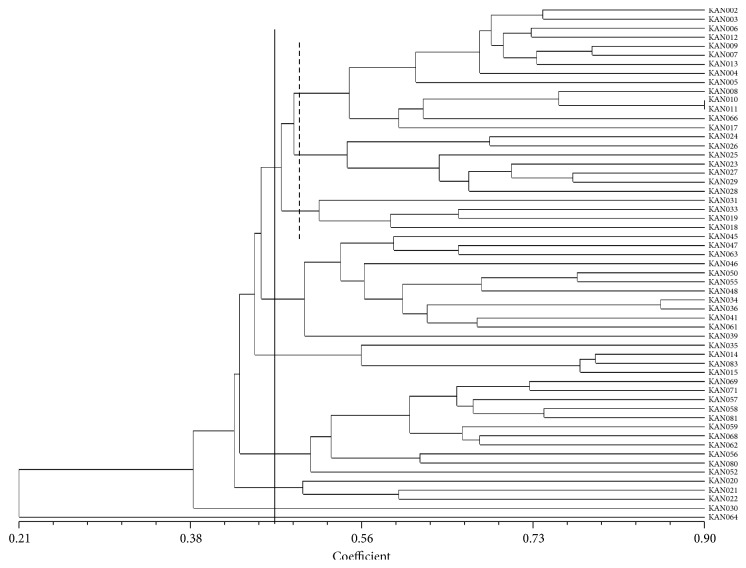
Dendogram based on UPGMA, representing the genetic relationship among the* E. elatior* accessions using ISSR markers.

The genetic relationship of 57 torch ginger accessions was obtained from the scoring data using Dice similarity coefficient. Cluster analysis was conducted to group genotypes into a dendrogram. The dendrogram was constructed using Dice similarity coefficient. The 57 accessions were grouped into seven major clusters at a coefficient value of 0.47 (Figure 1) and the similarity coefficient value ranged from 0.21 to 0.90. Cluster I comprised 24 accessions, which was divided into three subclusters (A, B, and C) which contain 13, 7, and 4 accessions, respectively. Cluster II consisted of 12 accessions; cluster III contained 4 accessions; cluster IV contained 11 accessions; cluster V consisted of 3 accessions, while both cluster VI and cluster VII are solitary (Table 8).

#### 3.1.3. Principal Component Analysis (PCA) of ISSR

**Figure 2:**
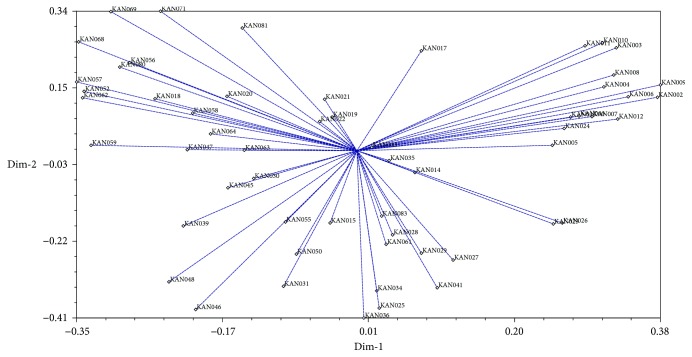
Two-dimensional plots of PCA indicating relationship among 57 torch ginger accessions based on ISSR markers.

The PCA analysis was performed to determine the dissimilarity of the accession and to verify the clustering pattern from the dendrogram. Based on PCA analysis, 57 accessions were also grouped into seven groups as shown in the two-dimensional (2D) plots (Figure 2). The results of clustering in the dendrogram and PCA analysis are consistent with each other.

### 3.2. SSR Analysis

Six primers were chosen based on their ability to generate polymorphic and scorable amplification products. High polymorphism was observed in all the six amplified loci, Eela2, Eela4, Eela5, Eela17, Eela18, and Eela21. A total of 363 alleles from six SSR markers were distinguished across all 57 accessions. The number of alleles per primer pair (locus) ranged from 54 (Eela2 and Eela4) to 70 for Eela17 with an average of 60.5 (Table 9). PIC value is a measure of the allelic differentiation. The highest PIC value was observed for the marker Eela17 (0.98) and the lowest (0.92) for the marker Eela5 with the average PIC value of 0.95. This study discovered that the allele frequency was low (< 0.30) in all primers with the mean of 0.14. Genetic diversity showed the range from 0.92 (Eela5) to 0.98 (Eela17) with an average of 0.96. The heterozygosity among the six amplified markers was high. All markers showed heterozygosity ranging between 0.45 (Eela2) and 0.81 (Eela17) with an average of 0.66.

The genetic distance between torch ginger accessions is displayed in Table 10. The values ranged from 0.04 (between Perak and Terengganu populations) to 0.25 (between Kelantan and Melaka populations).

#### 3.2.1. Cluster Analysis of SSR

**Figure 3:**
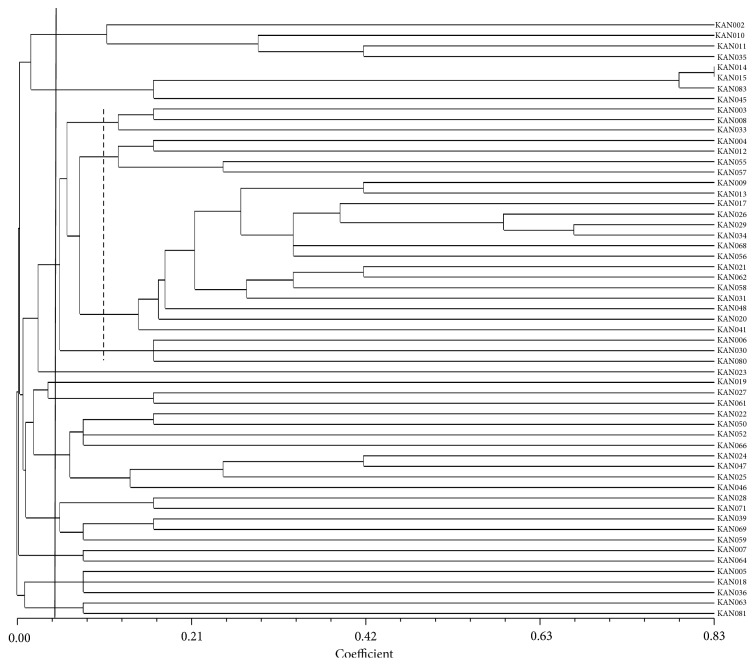
Dendrogram showing diversity among 57 accessions of torch ginger based on SSR markers.

Similarity matrices of all the 57 accessions were generated using NTSYS-pc version 2.10. The allelic diversity data was used to produce a dendrogram to explain the genetic relationships among the accessions (Figure 3). All genotypes were grouped into 11 clusters at 0.04 coefficient. Cluster III was the largest group, consisting of 25 accessions (Table 11). This cluster was further divided into four subclusters; “A” contains three accessions, “B” four accessions, “C” 15 accessions, and “D” three accessions. Cluster VII had the second highest number (8) of accessions followed by cluster VIII which contained 5 accessions, clusters I and II were comprised of four accessions, respectively, and cluster X contained three accessions. Clusters VI, IX, and XI individually consisted of two accessions and the single accession constructed both cluster IV and cluster V.

#### 3.2.2. Principal Component Analysis (PCA) of SSR

**Figure 4:**
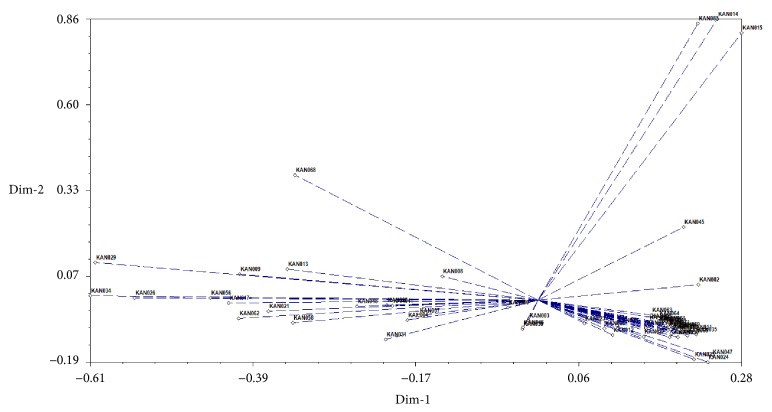
Two-dimensional PCA representing relationships among 57 torch ginger accessions based on SSR markers.

A principal coordinate analysis was presented as a complementary idea of the relationships among torch ginger accessions (Figure 4). The result roughly corresponds to the cluster dendrogram.

### 3.3. Relationship of Nei's Genetic Distance between ISSR and SSR Markers

The values of Nei's genetic distance among seven states based on ISSR markers were low compared to SSR markers (Table 12). However, all the pairwise values had no significant difference to each other and displayed a slightly high genetic distance. The pair of torch ginger accessions from Perak and Terengganu revealed the most distinct relationship with the value of 0.02 by ISSR and 0.04 by SSR markers.

## 4. Discussions

The evaluation of genetic diversity is crucial for the effective management and conservation of available genetic variability. The discovery of genetic dissimilarities and the interpretation of genetic associations among genotypes are very important for species protection and the sustainable use of plant genetic resources [16]. Molecular characterizations have been the favored selection criteria for breeding as they are more consistent, reliable, and less affected by environmental variations [41]. Since the phenotyping traits are commonly influenced by the environment, a number of molecular markers have been employed to explore the degree of variation, relationships, and genetic structure of plant genetic resources.

In the most recent years, many researches revealed that PCR-based techniques (SSR and ISSR) are effective to study the relationship or diversity between different species. Predominantly, they have been used to study the similarity between different varieties of ginger. In the present study, both ISSR and SSR markers were effective to characterize and differentiate between the accessions of torch ginger. They were capable of detecting the polymorphic and monomorphic alleles of accessions. Moderate-to-high level of genetic diversity among the torch ginger accessions was detected by ISSR and SSR markers.

In ISSR, all screened primers resulted in 11% polymorphism. These primers were dinucleotide repeats and this is in line with earlier report which discovered high polymorphic potential of dinucleotide [42] through their studies using ISSR in ginger cultivars. Generally, the selected ISSR primers produced an average of 17.9 bands with 1.43 effective alleles per locus (Table 4). Based on each locus, the values were much higher than those reported by Das* et al.* [43] and Pandotra* et al.* [42, 44] for various ISSR loci in ginger species with the average of 3-7, 7-17, and 6-18 bands per locus, respectively. However, the value revealed by this study is smaller than that described by Mohanty* et al.* [45] who detected 18.4 for the average of 16-22 bands per locus in ten species of Zingiberaceae. The differences between studies probably depend on the primer used and the band location. Thus, it is necessary to assure the same location of bands detected when repeating the experiments.

The use of ISSR primers is considered highly informative with the PIC value more than 0.30 (Table 4). However, SSR markers were revealed to be more informative compared to ISSR with the PIC value greater than 0.9. Effectiveness of a marker depends on the information content and the number of markers produced individually [46].

The ISSR markers also displayed various levels of genetic diversity among torch ginger accessions. Average value (0.41) of Shannon's information index (Table 5) was comparable to the value (0.4324) reported by Taheri* et al.* [47] for* Curcuma* varieties. Besides that, the range of Nei's gene diversity and its average (0.26; Table 5) also is in agreement with the findings of Taheri* et al.* [47] who found the average to be 0.2901. This average value is smaller than the value reported by Das* et al.* [43] who discovered 1.44 in ten* Zingiber moran* ecotypes.

Genetic polymorphism analysis in different populations also showed various levels of genetic diversity. Based on the (I) and (H_e_) values, population from Kelantan and Melaka showed a slightly lower genetic diversity. Meanwhile, three populations, namely, Pahang (0.31), Terengganu (0.30), and Perak (0.30), presented the highest genetic heterogeneity through Shannon's information index (Table 6). This highly detected polymorphism is in line with the findings of Singh* et al.* [48] who reported 0.3775 on turmeric population in different agroclimatic regions. This result signifies better adaptation of accessions to different range of environmental factors. The matrix of the pairwise population for Nei's genetic distance (Table 7) also revealed the genetic differentiation among the 57 torch ginger accessions from seven states. Interestingly, the populations from Perak and Terengganu were much closer despite the great distance between them. These values of Nei's genetic distance were low compared to other plants of different families such as* Portulaca oleracea *[49], in which a comparatively greater genetic distance was reported.

Furthermore, using the ISSR marker, the accessions could be broadly grouped into seven clusters (I-VII) and one of the major clusters (I) was further classed into smaller subclusters (Table 8). The accessions from different states were found to cluster together indicating no correlation between molecular groupings and their geographical origin. Similar observation was also made by Ranjbarfard* et al. *[50] to lemba (*Curculigo latifolia*) populations in Peninsular Malaysia. However, this result contradicts the report by Noroozisharaf* et al.* [51] on* Primula* and Panahi and Neghab [52] on safflower. The genotype similarities in the same cluster also might be influenced by participating a common lineage, convergent evolution, and selection of superior genotypes by farmers [52].

SSR primers such as Eela17, Eela18, and Eela21 showed polymorphism in all accessions studied with heterozygosity values above 70% (0.81, 0.72, and 0.71, resp.) (Table 9). Therefore, this indicates effectiveness and ability to detect polymorphisms in torch ginger accessions. More than 70% of heterozygosity value is considered more informative, reliable, and precise for population study. Number of alleles and their frequency in the population will influence the heterozygosity of markers [53]. PIC value is a measurement of allelic variation. All SSR markers had PIC value greater than 0.9 and thus were the most informative for differentiating among the torch ginger accessions. The highly polymorphic alleles might be related to the large genome size, outcrossing nature, or heterozygosity of the species [16]. The primer Eela17 had the highest PIC (0.98) with 54 alleles, and the major allele frequency was 0.06. PIC value was considered by number of alleles and their frequency [54]. Thus, one or two alleles that have high frequency will influence the low PIC values. In torch ginger accessions, the lowest PIC (0.92) was apparent in primer Eela5 with 59 alleles and 25% major allele frequency. The dissemination of the allele frequency at a single locus differs among the genotypes [55]. The allele frequency revealed by SSR markers for torch ginger accessions was quite low compared to other species such as soybean [18] and almond [56]. Genetic diversity displayed the possibility of two randomly chosen alleles to differ from the population. The genetic diversity values ranged from 0.92 to 0.98 with an average of 0.96 (Table 9). This average value was higher than the value recorded by Bisen* et al.* [18] in soybean genotypes, which is 0.2339. The utilization of SSR marker might be a pertinent factor in assessing genetic diversity. Vast genetic diversity might be influenced by the high levels of polymorphism discovered by the SSRs [16]. Since greater diversity was indicated by the higher value of genetic diversity parameter, SSR marker was the suitable choice for evaluation of genetic diversity in torch ginger germplasm collections.

Nei's genetic distance among seven states was also estimated using SSR markers to determine the level of population difference among torch ginger accessions. Nei's pairwise genetic distance among torch ginger accessions showed variable genetic distances (Table 10). The populations from Perak and Terengganu have shown lower genetic distance compared to other states indicating that they are genetically similar. Likewise, the other states displayed lower genetic distance compared to each other except those from Melaka which showed moderate genetic distance compared to other four populations, namely, Johor, Kedah, Kelantan, and Pahang. This could be due to the unique characters of Melaka population which the other populations might not have. Genetic distances data constructed by SSR markers should be preferred for generating selectable genetic variation from distant heritably genotypes [41].

Besides that, SSR markers through UPGMA cluster analysis give a better explanation on the association among 57 torch ginger accessions. All tested accessions were grouped into 11 main clusters based on the dendrogram. The coefficient of similarity index showed most of the accessions shared 0 to 0.8 index value, indicating that the accessions were highly different in their genetic characteristics. Two accessions (KAN014 and KAN015) showed a very close relationship in cluster II, suggesting that these two accessions were highly similar in their genetic characteristics. This is due to the fact that they were collected from the same district (Terengganu). However, most of the accessions from seven states were clustered separately and part of those was often mixed with others from different state. The results emphasized that genetic diversity among torch ginger accessions is not influenced by their geographical origin. Besides that, same state could be different in terms of environmental characteristic within it. On the other hand, this indicated little or no location specificity among the torch ginger accessions. This finding also is in agreement with Distefano* et al.* [56], Liang* et al.* [57], and Wang* et al.* [58] who studied genetic diversity on different crops using SSR markers. Distefano* et al.* [56] suggested that the genetic variation not only relies on their geographical origin but also is likely the effect of human selection and distribution of the most valuable genotypes.

Consequently, this germplasm would enhance the local gene pool and offer additional information for crop improvement programs in the future [58]. Pradhan* et al.* [59] opined that genotypes that constructed dissimilar groups are potential germplasms that might be utilized for the genetic base broadening. Similarly, as a vegetatively propagated crop, torch ginger has high levels of heterozygosity and hybridization between genetically different genotypes and could broaden the genetic base for selection.

## 5. Conclusion

This is the first report on the assessment of genetic variation in* E. elatior* accessions using molecular markers in Malaysia. Genetic diversity revealed the existence of a considerable degree of genetic variation among accessions of torch ginger as discovered by ISSR and SSR markers. The detection of high polymorphism and resolving power of marker system used in this study are of substantial consequence. The amplification of large number of polymorphic bands suggested that the primer sets used in this study could be of significance for the measurement of genetic diversity in torch ginger accessions. The combination of different molecular markers offered reliable and convincing information about the genetic diversity of torch ginger germplasm. Based on data generated from the two molecular markers, SSR marker was more informative compared to ISSR in terms of gene diversity, PIC values, and heterozygosity. SSR also revealed high ability in evaluating diversity levels, genetic structure, and relationships of torch ginger due to their codominance and rich allelic diversity. Generally, this study provides an initial scientific basis of data for torch ginger improvement and conservation programs in the future.

## Figures and Tables

**Table 1:** List of torch ginger accessions and their origin.

Population	Accessions	Origin
Perak	KAN002	Kg. Bongor, Grik
	KAN003	Kg. Tersusun, Grik
	KAN005	Kg. Pasang Api, Bagan Datoh, Hilir Perak
	KAN006	Kg, Sg. Ular, Bagan Datoh, Hilir Perak
	KAN008	Kg. Paya, Changkat Jering
	KAN009	Kg. Jamuan Jenalik, Kuala Kangsar (A)
	KAN010	Kg. Jamuan Jenalik, Kuala Kangsar (B)
	KAN011	Kg. Seterus, Sauk, Kuala Kangsar
	KAN012	Kg. Kerian, Batu Kurau
	KAN013	Kg. Lalang, Grik
	KAN031	Kg. Serentang, Bidor, Batang Padang
	KAN045	Kg. Lempor Tengah, Kuala Kangsar
	KAN046	Kg. Pecah Batu, Kuala Kangsar
	KAN047	Lata Putih, Bukit Kulim, Larut Matang

Terengganu	KAN007	Kg, Telok, Kuala Nerus
	KAN014	Kg Tepus, Hulu Dungun
	KAN015	Kg. Pasir Raja, Hulu Dungun
	KAN017	Kg. Matang, Hulu Terengganu
	KAN018	Kg. Pasir Dula, Hulu Terengganu
	KAN019	Kg. Basong, Hulu Terengganu
	KAN020	Kg. Bahagia, Tok Kah, Kuala Abang, Dungun
	KAN021	Kg. Bemban, Dungun
	KAN022	Kg, Binjai, Kemaman
	KAN023	Kg. Che Long, Permaisuri, Setiu
	KAN024	Felda Selasih, Permaisuri, Setiu
	KAN025	Felda Selasih, Permaisuri, Setiu
	KAN026	Kg. Padang Bual, Pasir Akar, Jerteh
	KAN027	Kg, Pecah Rotan, Bt. Rakit, Kuala Terengganu
	KAN028	Mengabang Lekor, Bt. Rakit, Kuala Terengganu
	KAN029	Kg. Din Maras, Bt. Rakit, Kuala Terengganu
	KAN030	Chendering, Marang
	KAN059	Kg. Tebing Tembah, Paka, Dungun
	KAN083	Kg. Kuala Kubang, Jabi

Kedah	KAN004	Kg. Titi Terus, Yan
	KAN033	Kg. Tok Weng, Pulai, Baling
	KAN034	Kg. Telok Durian, Kupang, Baling
	KAN035	Kg. Bukit Iboi, Kupang, Baling
	KAN036	Kg. Parit Panjang, Kupang, Baling
	KAN039	Kg. Bt. 13.5, Kodiang, Jitra
	KAN041	Kg. Padang Gaung, Hulu Melaka, Pulau Langkawi

Johor	KAN052	Felda Ulu Dengar, Kluang
	KAN055	Compartmen 123, Hutan Simpan Labis, Segamat (A)
	KAN056	Compartmen 123, Hutan Simpan Labis, Segamat (B)
	KAN057	Hutan Rekreasi Gunung Arong, Mersing
	KAN058	Kg. Tenglu, Mersing
	KAN068	Kg. Sungai Mengkuang, Triang, Mersing

Pahang	KAN061	Kg. Peruas, Ulu Dong, Raub
	KAN062	Kg. Peruas, Ulu Dong, Raub
	KAN063	Kg. Peruas, Ulu Dong, Raub
	KAN064	Kg. Peruas, Ulu Dong, Raub
	KAN066	Taman Herba Jabatan Hutan Som, Ulu Cheka, Jerantut

Kelantan	KAN048	Kg. Jeli Dalam, Jeli
	KAN069	Kg. Kundur, Gua Musang
	KAN071	RPT Batu Mengkebang, Kuala Krai

Melaka	KAN080	Kg. Air Hitam Darat, Masjid Tanah
	KAN081	Kg, Permatang, Kuala Sungai Baru, Alor Gajah

**Table 2:** Profiles of selected ISSR primers used in this study.

No.	Primer	Primer sequence 5'-3'	Ta (°C)
1	UBC808	(AG)_8_C	52.5
2	UBC809	(AG)_8_G	52.9
3	UBC811	(GA)_8_C	52.5
4	UBC817	(CA)_8_A	51.7
5	UBC830	(TG)_8_G	52.2
6	UBC855	(AC)_8_YT	52.4
7	UBC859	(TG)_8_RC	54.7
8	UBC880	GGAGAGGAGAGGAGA	53.1
9	UBC885	BHB(GA)_7_	51.2
10	UBC888	BDB(CA)_7_	51.4
11	UBC891	HYH(TG)_7_	53.7

**Table 3:** Details of SSR primers used in this study.

No.	Primer	Primer sequence 5'-3'	Allele size (bp)	Repeat unit
1.	Eela2	F CACGACGTTGTAAAACGACGCGCGGACTAACTGTTCAT	150	(AC)_7_(CT)_9_(TA)_6_
		R GACAAGACCACGACCGTATT		
2.	Eela4	F CACGACGTTGTAAAACGACAGGGACAAAGAACAGGAACC	231	(TG)_7_
		R GCAACAGGCATTGTCCTAAG		
3.	Eela5	F CACGACGTTGTAAAACGACAGTCAGACACTTGGCAGCTC	203	(AC)_13_
		R TAGACTGAGATCGCCGAAAG		
4.	Eela17	F CACGACGTTGTAAAACGACCGGACTAGCTTCGACAATGA	214	(GA)_16_
		R GGAGGAGGGTTTCTTATTCG		
5.	Eela18	F CACGACGTTGTAAAACGACCTTGAGAGATCGGACGACAA	249	(AC)_9_
		R CCGGTAGGAAATTCACGTAG		
6.	Eela21	F CACGACGTTGTAAAACGACCCAAGGGTACAACACACACA	170	(AG)_9_
		R CGAATTCCACTAGGGGTTCT		

**Table 4:** Amplified products of 57 accessions *E. elatior* from ISSR analysis.

Primer	TB	PB	PPB (%)	Amplicon band size (bp)	PIC	EMR	MI	RP
UBC808	20	20	100	300-2000	0.35	13.30	4.60	8.90
UBC809	15	15	100	450-1900	0.46	20.93	9.59	10.66
UBC811	13	13	100	300-1350	0.33	12.31	4.11	5.51
UBC817	14	14	100	400-4000	0.37	14.79	5.53	6.97
UBC830	17	17	100	450-3000	0.32	11.88	3.83	6.86
UBC855	21	21	100	300-3000	0.32	11.90	3.79	8.34
UBC859	18	18	100	320-2500	0.33	12.56	4.15	7.52
UBC880	17	17	100	320-2500	0.40	16.35	6.52	9.34
UBC885	30	30	100	290-2050	0.33	12.43	4.16	12.72
UBC888	11	11	100	400-1600	0.50	28.73	14.35	10.66
UBC891	21	21	100	350-2000	0.32	11.95	3.84	8.45

Total	197	197						
Mean	17.9	17.9	100		0.37	15.19	5.86	8.72

*Note.* TB: total number of bands; PB: polymorphic band; PPB (%): percentage of polymorphic band (%); PIC: polymorphic information content; EMR: effective multiplex ratio; MI: marker index; and RP: resolving power of primer.

**Table 5:** Genetic diversity parameter of the amplified ISSR markers in *E. elatior*.

Locus/primer	Observed no. of alleles per locus (na)	Effective no. of alleles per locus (ne)	Nei's gene diversity (h)	Shannon's index (I)
UBC808	2	1.40	0.26	0.40
UBC809	2	1.46	0.27	0.41
UBC811	2	1.36	0.22	0.35
UBC817	2	1.54	0.31	0.47
UBC830	2	1.36	0.23	0.37
UBC855	2	1.41	0.24	0.38
UBC859	2	1.42	0.26	0.40
UBC880	2	1.48	0.29	0.45
UBC885	2	1.44	0.28	0.44
UBC888	2	1.50	0.30	0.47
UBC891	2	1.38	0.24	0.39

*Mean*	*2*	*1.43*	*0.26*	*0.41*

St.	0	0.33	0.16	0.21

**Table 6:** Genetic diversity parameter in *E. elatior* accessions as detected by ISSR markers.

Population	N	Na	Ne	I	He	uHe	% Polymorphism
Perak	15	1.34	1.31	0.30	0.19	0.20	0.66
Kedah	7	1.16	1.26	0.26	0.16	0.18	0.56
Terengganu	19	1.59	1.30	0.30	0.19	0.20	0.79
Kelantan	3	0.79	1.23	0.19	0.13	0.16	0.35
Johor	6	0.92	1.23	0.21	0.14	0.15	0.44
Pahang	5	1.25	1.34	0.31	0.20	0.23	0.62
Melaka	2	0.58	1.17	0.14	0.10	0.13	0.24

Mean	8.14	1.09	1.26	0.24	0.14	0.16	0.52

*Note.* N: number of accessions; Na: number of alleles per locus; Ne: number of effective alleles per locus; I: Shannon's index; He: expected heterozygosity; uHe: unbiased expected heterozygosity.

**Table 7:** Pairwise population matrix of Nei's genetic distance.

States	Perak	Kedah	Terengganu	Kelantan	Johor	Pahang
Kedah	0.03					
Terengganu	0.02	0.03				
Kelantan	0.07	0.08	0.07			
Johor	0.05	0.06	0.04	0.05		
Pahang	0.04	0.05	0.03	0.08	0.05	
Melaka	0.08	0.09	0.07	0.07	0.05	0.08

**Table 8:** Accessions comprising various clusters as shown in the dendrogram based on UPGMA.

Clusters	Accessions
Cluster I	A: KAN002, KAN003, KAN006, KAN012, KAN009, KAN007, KAN013, KAN004, KAN005, KAN008, KAN010, KAN011, KAN066, KAN017
	B: KAN024, KAN026, KAN025, KAN023, KAN027, KAN029, KAN028,
	C: KAN031, KAN033, KAN019, KAN018
Cluster II	KAN045, KAN047, KAN063, KAN046, KAN050, KAN055, KAN048, KAN034, KAN036, KAN041, KAN061, KAN039
Cluster III	KAN035, KAN014, KAN083, KAN015
Cluster IV	KAN069, KAN071, KAN057, KAN058, KAN081, KAN059, KAN068, KAN062, KAN056, KAN080, KAN052
Cluster V	KAN020, KAN021, KAN022
Cluster VI	KAN030
Cluster VII	KAN064

**Table 9:** Genetic diversity parameter of the amplified SSR markers in *E. elatior.*

Marker	Major allele frequency	Number of genotypes	Number of alleles	Gene diversity	Heterozygosity	PIC
Eela2	0.09	46.00	54.00	0.97	0.45	0.97
Eela4	0.15	44.00	54.00	0.96	0.67	0.95
Eela5	0.26	44.00	59.00	0.92	0.59	0.92
Eela17	0.06	52.00	70.00	0.98	0.81	0.98
Eela18	0.09	47.00	64.00	0.97	0.72	0.96
Eela21	0.18	46.00	62.00	0.95	0.71	0.95

Mean	0.14	46.50	60.50	0.96	0.66	0.95

**Table 10:** Pairwise population matrix of Nei's genetic distance among seven states using SSR markers.

States	Johor	Kedah	Kelantan	Melaka	Pahang	Perak
Kedah	0.09					
Kelantan	0.19	0.15				
Melaka	0.23	0.20	0.25			
Pahang	0.13	0.11	0.17	0.20		
Perak	0.09	0.06	0.13	0.16	0.08	
Terengganu	0.08	0.06	0.13	0.16	0.08	0.04

**Table 11:** Accessions comprising various clusters as shown in the dendrogram based on UPGMA method using SSR markers.

Clusters	Accessions
Cluster I	KAN002, KAN010, KAN011, KAN035
Cluster II	KAN014, KAN015, KAN083, KAN045
Cluster III	A: KAN003, KAN008, KAN033 B: KAN004, KAN012, KAN055, KAN057 C: KAN009, KAN013, KAN017, KAN026, KAN029, KAN034, KAN068, KAN056, KAN021, KAN062, KAN058, KAN031, KAN048, KAN020, KAN041 D: KAN006, KAN030, KAN080
Cluster IV	KAN023
Cluster V	KAN019
Cluster VI	KAN027, KAN061
Cluster VII	KAN022, KAN050, KAN052, KAN066, KAN024, KAN047, KAN025, KAN046
Cluster VIII	KAN028, KAN071, KAN039, KAN069, KAN059
Cluster IX	KAN007, KAN064,
Cluster X	KAN005, KAN018, KAN036
Cluster XI	KAN063, KAN081

**Table 12:** Pairwise population matrix of Nei's genetic distance among seven states based on ISSR and SSR (bold) markers.

States	Perak	Kedah	Terengganu	Kelantan	Johor	Pahang	Melaka
Perak	1	**0.06**	**0.04**	**0.13**	**0.09**	**0.08**	**0.16**
Kedah	0.03	1	**0.06**	**0.15**	**0.09**	**0.11**	**0.20**
Terengganu	0.02	0.03	1	**0.13**	**0.08**	**0.08**	**0.16**
Kelantan	0.07	0.08	0.07	1	**0.19**	**0.17**	**0.25**
Johor	0.05	0.06	0.04	0.05	1	**0.13**	**0.23**
Pahang	0.04	0.05	0.03	0.08	0.05	1	**0.20**
Melaka	0.08	0.09	0.07	0.07	0.05	0.08	1

## Data Availability

The data used to support the findings of this study are available from the corresponding author upon request.
